# IHRAS: Automated Medical Report Generation from Chest X-Rays via Classification, Segmentation, and LLMs

**DOI:** 10.3390/bioengineering12080795

**Published:** 2025-07-24

**Authors:** Gabriel Arquelau Pimenta Rodrigues, André Luiz Marques Serrano, Guilherme Dantas Bispo, Geraldo Pereira Rocha Filho, Vinícius Pereira Gonçalves, Rodolfo Ipolito Meneguette

**Affiliations:** 1 Department of Electrical Engineering, University of Brasilia, Federal District, Brasília 70910-900, Brazil; andrelms@unb.br (A.L.M.S.); guilherme.bispo@redes.unb.br (G.D.B.); geraldo.rocha@uesb.edu.br (G.P.R.F.); vpgvinicius@unb.br (V.P.G.); 2 Institute of Mathematical and Computer Sciences, University of São Paulo, São Carlos 13566-590, Brazil; meneguette@icmc.usp.br; 3 Department of Exact and Technological Sciences, State University of Southwest Bahia, Vitória da Conquista 45083-900, Brazil

**Keywords:** classification, deep learning, LLM, pathology, radiology, segmentation

## Abstract

The growing demand for accurate and efficient Chest X-Ray (CXR) interpretation has prompted the development of AI-driven systems to alleviate radiologist workload and reduce diagnostic variability. This paper introduces the Intelligent Humanized Radiology Analysis System (IHRAS), a modular framework that automates the end-to-end process of CXR analysis and report generation. IHRAS integrates four core components: (i) deep convolutional neural networks for multi-label classification of 14 thoracic conditions; (ii) Grad-CAM for spatial visualization of pathologies; (iii) SAR-Net for anatomical segmentation; and (iv) a large language model (DeepSeek-R1) guided by the CRISPE prompt engineering framework to generate structured diagnostic reports using SNOMED CT terminology. Evaluated on the NIH ChestX-ray dataset, IHRAS demonstrates consistent diagnostic performance across diverse demographic and clinical subgroups, and produces high-fidelity, clinically relevant radiological reports with strong faithfulness, relevancy, and alignment scores. The system offers a transparent and scalable solution to support radiological workflows while highlighting the importance of interpretability and standardization in clinical Artificial Intelligence applications.

## 1. Introduction

X-rays are the most common imaging technique used in medical diagnostics due to their widespread availability, speed, and cost-effectiveness [1]. Specifically, chest X-rays (CXRs) are useful diagnostic imaging modalities in clinical practice, which aid medical professionals in detecting and monitoring of a wide range of thoracic diseases. Interpreting these images, however, requires radiological expertise and time, and remains susceptible to inter-reader variability [2].

In addition to this, the high volume of CXR studies contributes to raising a radiologist workload [3], increasing the risk of reporting delays and potential oversights, particularly for subtle findings. In resource-limited locations, the shortage of specialized radiologists further complicates timely and accurate diagnoses. These challenges indicate the need for improved solutions to enhance the efficiency, accuracy, and accessibility of CXR medical report generation [4].

To address these challenges, deep learning systems learn to recognize patterns in medical images, helping detect pathologies like lung and heart diseases [5]. Instead of relying solely on human experts, the technology can quickly analyze a large volume of X-rays, flagging potential issues for physicians to review.

It is, however, important that the Artificial Intelligence (AI) can also highlight the concerning areas on the image, making it easier for medical professionals to understand its findings, enhancing the clearer communication between the AI system and medical professionals and strengthening the trust in the model [6].

Despite the progress in AI-driven CXR interpretation, existing solutions often lack a full integration of visual explanation, anatomical relevance, and structured report generation, limiting their clinical adoption.

In this study, we introduce the Intelligent Humanized Radiology Analysis System (IHRAS), a modular architecture designed to overcome limitations in chest X-ray interpretation. IHRAS integrates disease classification, visual explanation, anatomical segmentation, and automated medical report generation. Upon receiving a chest X-ray image, the system classifies it into 14 common thoracic diseases using a deep Convolutional Neural Network (CNN). To enhance interpretability, it uses Gradient-weighted Class Activation Mapping (Grad-CAM) to highlight the spatial regions most relevant to the identified conditions. To enhance interpretability, We further segment these regions using a dedicated anatomical segmentation model, assigning them to clinically relevant anatomical structures. Ultimately, these findings are used to condition a Large Language Model (LLM) that generates a human-readable medical report using the Systematized Nomenclature of Medicine—Clinical Terms (SNOMED CT). IHRAS is tested on the NIH CXR dataset, due to its clinical relevance, diversity and standardization.

While IHRAS demonstrates the possibility of automating radiological workflows, auditability is necessary to maintain accountability. The system’s decisions must be interpretable and traceable to allow clinicians to validate and understand AI outputs, mitigating the risk of over-reliance on automated systems.

Another consideration is the generalizability of the proposed system across diverse populations and imaging conditions. CXR datasets often reflect demographic and geographic biases that may lead to suboptimal performance in underrepresented groups [7]. Therefore, an evaluation across multi-institutional datasets with diverse patient profiles is necessary to ensure consistent performance.

### 1.1. Contributions and Limitations of the Work

The primary contributions of this work lie in the development of a modular architecture for automated chest X-ray report generation that unifies classification, localization, segmentation, and language modeling. The system utilizes Grad-CAM to produce visual explanations of disease predictions, which are further enhanced by anatomical context through segmentation, thereby improving both interpretability and clinical relevance. This integrated framework represents a significant step forward in radiological image analysis and report generation.

Unlike prior architectures, IHRAS integrates classification, spatial reasoning, anatomical segmentation, and structured language modeling into a unified pipeline, thus providing a more complete approach.

Due to its modular nature, IHRAS may be adapted to generate medical reports beyond CXR, including X-rays of other body parts, such as abdomen and head, as well as other imaging modalities, such as blood cell microscopy and ocular imaging.

As limitation, the pretrained model achieved a modest F1-score for the multilabel classification problem, which, while comparable to other generalist models in the literature, reflects the challenge of diagnosing diverse pathologies simultaneously.

Furthermore, although IHRAS employs Grad-CAM visualizations to provide interpretable predictions, the lack of pixel-level annotations in the dataset prevents quantitative validation of the highlighted regions. Consequently, the accuracy of the attention maps cannot be formally assessed.

### 1.2. Organization of the Work

The remainder of this paper is structured as follows. Section 2 reviews the related literature. Section 3 presents the proposed modular architecture of IHRAS, whilst Section 4 discusses the results obtained with the approach. Section 5 concludes the paper.

## 2. Related Works

Due to the significant relevance and growing demand for advancements in chest and general X-ray research, several datasets have been published to support studies in this field [8], including in specific regions, such as in Brazil [9]. These datasets facilitate the development and validation of machine learning models, diagnostic tools, and other medical imaging applications.

### 2.1. Medical Classification Models

Using such datasets, CNNs and transformer-based architectures have demonstrated high accuracy in detecting medical conditions such as lung cancer, tuberculosis and COVID-19 [10]. Our work assesses different CNN models with the NIH CXR dataset, selecting the model and its parameters that optimize the classification metrics to generate medical reports.

As an example, ref. [11] have achieved an F1-score of 0.937 and of 0.954 in different datasets for the detection of pneumonia using VGG16 with Neural Networks. These results are significant due to the clinical importance of rapid and accurate detection of this infection, which represents a significant cause of hospitalization worldwide.

While these advancements in deep learning for medical imaging are promising, the reliance on centralized datasets raises concerns about data privacy and security, as patient confidentiality in the healthcare domain is fundamental. Federated Learning (FL) addresses these challenges by enabling collaborative model training across multiple institutions without sharing raw data, thus preserving privacy [12]. In the context of chest X-ray analysis, FL can leverage diverse datasets from different hospitals to improve model generalizability, promoting the diagnosis of diseases while complying with data protection regulations [13].

Deep Learning models are applicable in several healthcare domains beyond the scope of X-ray images. For instance, ref. [14] presented a deep transfer learning framework for the automated classification of leukemia subtypes using microscopic peripheral blood smear images. They used a dataset comprising 1250 images across five categories of the disease, with feature extraction performed via fine-tuned VGG16 and classification using Support Vector Machine (SVM) and Random Forest. Despite achieving an accuracy of 84%, the study did not employ anatomical segmentation or language-based report generation. In contrast, our proposed IHRAS system inegrates pathology classification with anatomical segmentation and natural language report generation, enhancing clinical utility.

### 2.2. Explainable Artificial Intelligence and Image Segmentation

In addition to an accurate pathology classification, ref. [15] indicates that an explainable Artificial Intelligence (XAI) enhances the transparency, reliability, and safety of diagnostic systems, contributing to improved healthcare delivery. The work [16] categorizes XAI techniques into four critical dimensions, namely data, model, post-hoc, and evaluation. In healthcare, where misdiagnoses may have great impact, XAI must justify predictions.

Several XAI methods are available [17]. However, unlike purely statistical XAI methods, such as Shapley Additive Explanations, Grad-CAM generates heatmaps for spatial reasoning, a desired characteristics for trust and adoption in time-sensitive diagnostics.

In the medical field, Grad-CAM has been adopted to visualize the regions of an image that most influenced the model’s decision, providing explainability into the basis of its predictions [18,19]. Because of this, IHRAS adopts Grad-CAM, which provides interpretable visual explanations of model decisions, allowing clinicians to understand the AI’s reasoning for the detected abnormalities.

While Grad-CAM identifies areas influencing a model’s decision, segmentation models transform these heatmaps into medical terms [20]. This terminology alignment enables radiologists to rapidly verify AI findings against anatomical expectations. Also, structured reporting systems can auto-populate preliminary impressions with location-specific pathology descriptions.

To advance this, Ref. [21] have proposed an enhanced multiverse optimizer-based multilevel thresholding image segmentation method to improve COVID-19 chest radiograph analysis. By incorporating horizontal and vertical search mechanisms, it enhances global search capabilities and avoids local optima. The results highlight its potential as a reliable tool for medical institutions handling COVID-19 cases.

### 2.3. LLM for Medical Report Generation

Recent works have explored adapting LLMs to healthcare, where general-purpose models often struggle due to terminology gaps and limited task-specific training data. As an example, MedChatZH [22] proposed a decoder-based model trained on a curated corpus of Traditional Chinese Medicine literature, demonstrating improved performance over generic dialogue baselines, aligning with broader efforts to tailor LLMs for expert domains through targeted data augmentation and architecture adaptation. Their work highlights the effectiveness of LLMs in medical applications. Therefore, in this paper, we use LLM to generate structured medical reports using the SNOMED CT standardized terminology system.

To the best of our knowledge, this is the first study to combine classification, explainability, segmentation and medical report generation with LLM in the same architecture, as depicted in Table 1.

## 3. Methodology

**Figure 1:**
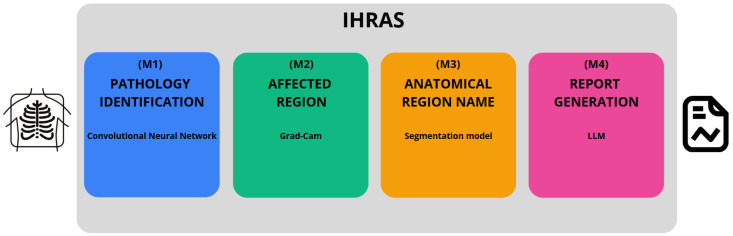
IHRAS architecture, and its four modules.

Our proposed architecture, developed in Python version 3.11.12, is depicted in Figure 1, encompassing from the input of a CXR image to the generation of the medical report.

The IHRAS framework consists of four sequential modules. The first module, M1 (see Figure 1), detects abnormalities in the CXR and is detailed in Section 3.2. Modules M2 and M3, described in Section 3.3, localize the regions influencing the classification model’s decision and map them to their corresponding anatomical terms, respectively. Finally, M4 integrates the outputs from M1 and M3, using an LLM to generate the medical report, as discussed in Section 3.4.

### 3.1. The Dataset

The NIH CXR dataset, proposed by [29], is a publicly available dataset containing 112,120 chest X-ray images from 30,805 unique patients, annotated with up to 14 thoracic pathology labels derived from associated radiology reports. It is chosen due to its large scale, multi-label annotations, and open accessibility, which enables reproducible evaluation.

To reduce computational costs and processing time while maintaining a representative sample of the dataset for the models comparison, we employed a stratified random sampling strategy. The population was partitioned into homogeneous strata based on four clinically relevant variables: (i) patient age (grouped into 25-year intervals); (ii) radiological findings; (iii) patient gender; and (iv) radiographic view position.

The sample size per stratum is determined based on Equation (1), in which *Z* represents the Z-score, corresponding to the desired confidence level; *p* denotes the estimated proportion; and *e* stands for the margin of error. The variable *n* represents the sample size, whereas *N* is the population size.

(1) $$n=\frac{N\cdot Z^{2}\cdot p\cdot \left(1-p\right)}{e^{2}\left(N-1\right)+Z^{2}\cdot p\cdot \left(1-p\right)}$$

We selected 370 samples per stratum to achieve a 95% confidence level with a 5% margin of error for the largest strata (*N* = 9677). For smaller strata with fewer than 370 samples, all available samples were included. This stratified sampling approach preserves the original dataset’s clinical and demographic diversity. This results in a total of 50,133 selected X-ray images.

### 3.2. Pathology Identification (M1)

Each inputted X-ray image may be classified as according to different pathologies, namely atelectasis, cardiomegaly, consolidation, edema, effusion, emphysema, fibrosis, hernia, infiltration, mass, nodule, pleural thickening, pneumonia, and pneumothorax. It is a multi label classification problem, that is, each image can be associated with multiple labels simultaneously.

Three pretrained deep learning models are evaluated, and the best performing model is selected for integration into the IHRAS framework, to ensure optimal diagnostic accuracy. These models are obtained from TorchXRayVision [30], with their default configurations, and refer to a Densely Connected Convolutional Networks (DenseNet) [31] trained on several CXR datasets; a DenseNet trained specifically on the NIH dataset; and a Residual Neural Network (ResNet) [32] trained on several chest X-ray datasets. These models are selected due to their high accuracy in pathology classification [33].

These models are evaluated based on some metrics. The first one is precision, which measures the proportion of correctly predicted positive instances among all predicted positives, as according to Equation (2), where TP, FP, TN, and FN denote True Positives, False Positives, True Negatives, and False Negatives respectively. This metrics should be considered when the cost of false positives is high.

(2) $$\mathrm{Precision}=\frac{TP}{TP+FP}$$

Recall, also known as sensitivity and defined by Equation (3), quantifies the ability to identify true positives over all positive instances. It is a relevant metric when missing positive cases is undesirable.

(3) $$\mathrm{Recall}=\frac{TP}{TP+FN}$$

The F1-score, determined by Equation (4), represents the harmonic mean of precision and recall.

(4) $$F_{1}=2\times \frac{\mathrm{Precision}\times \mathrm{Recall}}{\mathrm{Precision}+\mathrm{Recall}}=\frac{2TP}{2TP+FP+FN}$$

Ultimately, specificity, calculated as in Equation (5), measures the ability to identify true negatives over all negative instances.

(5) $$\mathrm{Specificity}=\frac{TN}{TN+FP}$$

### 3.3. Affected Region and Its Anatomical Name (M2 and M3)

Since medical reports need to have clarity, Grad-CAM is used to enhance explainability, as it has been demonstrated to improve the interpretability of deep learning models in medical imaging [34]. This technique generates heatmaps that indicate the regions of the image that most strongly influenced the model’s diagnostic conclusions. This spatial alignment with radiological markers allows clinicians to audit whether the model’s decisions are anatomically plausible, complying with requirements for transparency in medical AI.

To further enhance the interpretability of the model’s decisions, a segmentation model is employed to identify the anatomical name of the regions highlighted by Grad-CAM. This ensures adherence to standardized medical reporting, as the medical report references meaningful structures rather than relying on generic image coordinates or non-clinical descriptors.

To achieve this segmentation, we use the Structure-Aware Relation Network (SAR-Net) model proposed by [35]. The SAR-Net model is evaluated on ChestX-Det, which is a subset of the NIH dataset, used in this work, achieving a Mean Intersection-Over-Union of 86.85%, outperforming comparable models [36,37,38]. The anatomical structures it is trained to identify are the aorta, facies diaphragmatica, heart, left clavicle, left hilus pulmonis, left lung, left scapula, mediastinum, right clavicle, right hilus pulmonis, right lung, right scapula, spine, and weasand.

### 3.4. Report Generation (M4)

The extracted diagnostic data, comprising identified pathologies with associated probabilities and affected anatomical regions, alongside supplementary inputs such as patient demographics (age, gender) and radiographic projection, is processed by an LLM to generate a medical report. This approach provides a patient-aware report generation.

To select the LLM model for this task, we considered the BRIDGE benchmark, that is composed of 87 tasks based on clinical data from real-world sources, covering nine languages [39]. The work compared a total of 52 different LLMs using multiple inference approaches, and DeepSeek-R1, an open-source model, achieved the highest score in radiology and ranks among the top 3 in the overall score, along with Gemini-1.5-Pro and GPT-4o. These general-purposed models achieved even better results than the medically fine-tuned ones. Because of these results, we use DeepSeek-R1 to generate the radiological report [40].

The report generation employs the CRISPE framework (Capacity, Role, Insight, Statement, Personality, Experiment) for structured prompt engineering, which enhances the LLM’s output quality [41]. This methodology ensures precise instruction by (C) defining the model’s clinical expertise boundaries; (R) establishing a radiologist role; (I) incorporating patient-specific contextual data and diagnosis; (S) specifying reporting requirements with SNOMED CT compliance; (P) maintaining professional tone consistency; and (E) limiting to a single response per prompt. The structure prompt following this framework is shown in Table 2.

SNOMED CT enhances this architecture with the standardization of clinical terminology, improving data quality through consistent indexing, and with the advance of the continuity of care through interoperable patient records. It also facilitates clinical research with a structured data aggregation, and improves patient safety [42]. Due to these benefits, the LLM is instructed to use SNOMED CT.

To evaluate the quality of the generated medical reports, we employed DeepEval [43], an open-source framework for assessing LLM outputs using an LLM-as-a-judge approach. The metrics considered in the assessment are faithfulness, to evaluate whether the generated report is aligned with the findings from the previous IHRAS steps; answer relevancy, to measure how relevant the report is in relation to the prompt; hallucination to detect unsupported claims; toxicity and bias, to identify harmful or discriminatory language; and prompt alignment to assess adherence to the CRISPE-structured instructions.

## 4. Results and Discussion

This section presents the results obtained with the IHRAS pipeline, from the CXR image input up to the report generation.

### 4.1. Classification Models Evaluation

For the disease classification module, we evaluate three models, namely a DenseNet trained on multiple CXR datasets, a DenseNet fine-tuned specifically on the NIH dataset, and a ResNet trained across diverse CXR datasets. The best-performing model, based on evaluation metrics, is selected for integration into IHRAS.

#### 4.1.1. Models Comparison

All evaluated models generate a probability score p∈[0,1] for each detectable pathology in the input X-ray image. To convert these continuous predictions into binary classifications (present/absent), an optimal decision threshold must be established.

**Figure 2:**
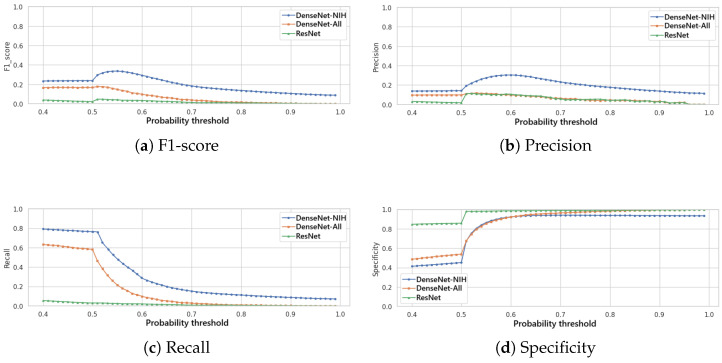
Comparison of evaluation metrics for different thresholds for the classification models.

Figure 2 presents the variation of the F1-score (Figure 2a), of the precision (Figure 2b), of the recall (Figure 2c) and of the specificity (Figure 2d) in function to the variation of this threshold for the three classification models. It is noted that the DenseNet trained specifically for the NIH dataset achieves the best F1-score, precision and recall for a given threshold, and, thus, this model is selected for the classification module of IHRAS in this work.

In this study, we employ the F1-score as our primary optimization metric, to balance false positives and false negatives. As seen in Figure 2a, the optimal decision threshold of 0.55 maximizes the F1-score at 0.34. This threshold reflects a balance of clinical priorities, equitably weighting sensitivity (to avoid missed diagnoses) and precision (to reduce false alarms). The obtained F1-score is comparable to those of other multilabel classification models evaluated on the same and other datasets, as shown in Table 3, especially considering the long-tailed characteristics of the used dataset [44].

Although the F1 score achieved by the classification model selected for IHRAS is lower than the best reported in [46], by Code-Free Deep Learning (CFDL) platforms, the chosen DenseNet offers significant advantages in terms of control and transparency, as users cannot audit the CFDL architecture and the explainability is hindered. Furthermore, the adopted DenseNet architecture outperformed all comparative studies in Table 3 in at least one performance metric.

In scenarios where false negatives incur higher costs, the threshold should be lowered to prioritize recall, ensuring fewer missed cases. Conversely, when false positives are more detrimental, the threshold should be raised to maximize precision.

**Figure 3:**
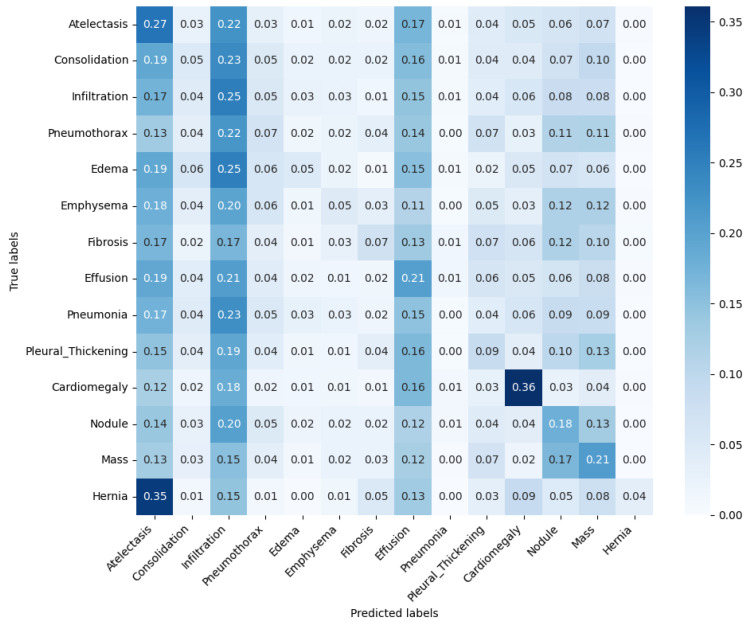
Normalized confusion matrix comparing model-predicted pathologies against true clinical diagnoses.

Figure 3 presents the normalized confusion matrix of the classification model, in which rows represent the true labels, while columns show predicted classifications. It indicates that the model tends to predict a CXR as atelectasis, infiltration and/or effusion, whilst labels such as pneumonia and hernia are more rarely predicted. This behavior possibly explains the modest F1-score obtained.

#### 4.1.2. Binary Classification

Although IHRAS is designed to identify and differentiate between several thoracic pathologies, we also evaluate its performance in a binary classification task distinguishing between abnormal and normal chest X-rays. In this binary setting, all images exhibiting at least one pathological finding, regardless of the specific diagnosis, are grouped under the abnormal class, whereas those explicitly labeled as no findings constitute the normal class. This evaluation enables the assessment of the model’s capability to serve as a general screening tool, flagging potentially abnormal cases for further review. It also provides insight into the system’s general sensitivity to pathological signals irrespective of specific diagnoses.

**Figure 4:**
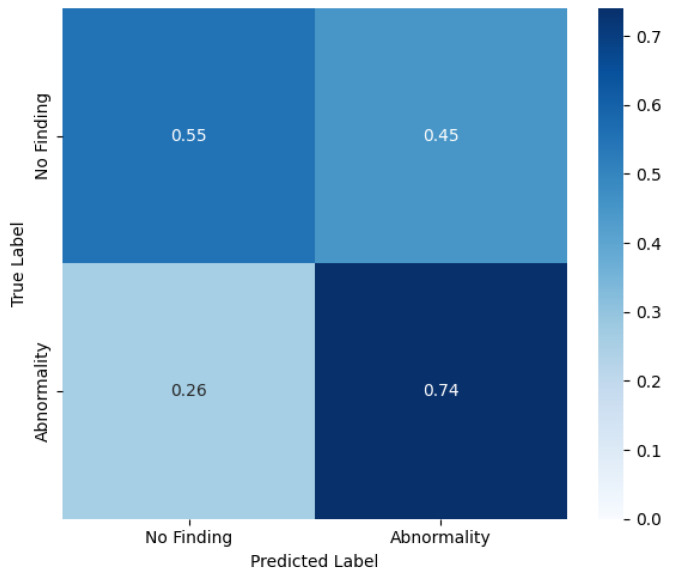
Normalized confusion matrix comparing for the binary classification.

In this binary classification, the same probability threshold of 0.55 is used, achieving a precision of 0.93, a recall of 0.74, a specificity of 0.55 and a F1-score of 0.83. The confusion matrix of this scenario is shown in Figure 4.

These results indicate the adaptability of IHRAS, demonstrating its capability to identify specific thoracic diseases and to function as a screening tool. This dual functionality broadens its potential applications in clinical workflows, from rapid triage to decision support.

#### 4.1.3. Sample Demographics

**Figure 5:**
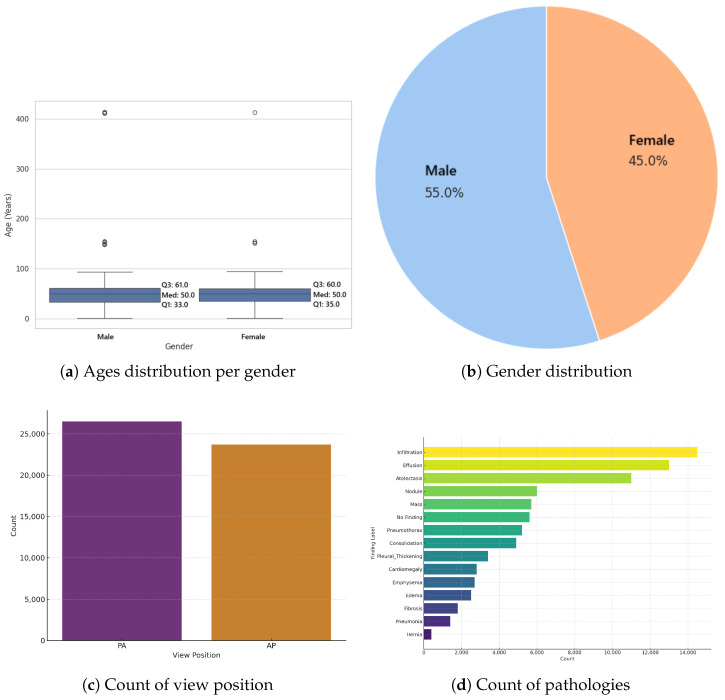
Properties of the X-ray images selected for the evaluation.

It has been observed by [7] that AI models underdiagnose pathologies in marginalized groups, concluding that there is a significant disparity when comparing “black female” patients with “white male”. To assess this disparity within the 0.55 threshold DenseNet model adopted by IHRAS, the demographic and clinical characteristics of the sampled dataset are presented in Figure 5.

Figure 5a reveals apparent anomalies in the age data, including patients recorded as over 400 years old. These outliers do not compromise the diagnosis, as age is not considered in the analysis, being only included as metadata in the report for informational purposes. The median age for both genders, however, is 50 years, suggesting these extreme outliers are likely artifacts of data entry errors rather than representative of the true distribution. The sampled dataset also contains slightly more males than females (Figure 5b) and Postero-Anterior (PA) views than Antero-Posterior (AP) (Figure 5c). Figure 5d show that the imbalance for the pathologies count is significantly greater, thus suggesting the long-tailed characteristics of the dataset. However, since this section focuses on assessing a pretrained model’s diagnostic performance across diverse subgroups, rather than training a new model, such imbalances reflect clinical variability rather than methodological limitations.

Upon comparing Figure 3 and Figure 5d, it is evident that the classification model exhibits a bias toward predicting the most frequently occurring pathologies in the dataset, such as infiltration, effusion, and atelectasis, while underrepresenting rarer conditions like pneumonia and hernia. This pattern suggests that the model’s predictions are influenced by class imbalance, which is a common challenge in classification problems in healthcare and in other domains [44,47,49].

The comparison of the evaluation metrics for these different demographics and clinical characteristics is presented in Table 4, which indicates no significant disparities between different demographic and clinical groups.

We quantify pairwise differences in model performance using Cohen’s *h* effect size measure, computed for all pairs of values within the same category. As shown in Table 5, the maximum observed *h* values across all categories is of 0.1003 (Findings/Recall), which is below Cohen’s threshold of 0.2 for small effects [50]. This demonstrates statistically negligible variation in model performance across the demographic subgroups, image acquisition parameters and clinical findings.

### 4.2. Report Generation Evaluation

The metrics associated to the generated medical reports are presented in Table 6. This evaluation assesses the LLM’s ability to generate accurate reports based on the classification results and on the identified affected anatomical region provided to it, rather than comparing against the actual annotated pathology. The performance of the disease classification model has been validated in Section 4.1.

Instead, this analysis tests the LLM’s capacity to faithfully translate input clinical data into coherent reports, to maintain contextual relevance, and to adhere to clinical reporting standards.

The results shown in Table 6 demonstrate capabilities for safe clinical deployment. They indicate that the IHRAS LLM module is capable of operating within the healthcare workflow, where accuracy, safety, and consistency are fundamental.

The faithfulness scores validate that reports accurately reflect diagnostic inputs, ensuring reliability in communicating critical findings, which is a fundamental requirement for medical decision-making. The strong performance in answer relevancy indicates that the reports have successfully fulfilled the prompt, while perfect hallucination, toxicity, and bias scores confirm the absence of fabricated claims and harmful content, addressing patient safety and ethical concerns. The prompt alignment results, though slightly lower than other metrics, still reflect robust adherence to structured clinical reporting standards.

These metrics, however, were obtained through an LLM-as-judge evaluation process, which, while efficient for automated assessment, introduces limitations regarding clinical validity.

### 4.3. IHRAS Case Studies

**Figure 6:**
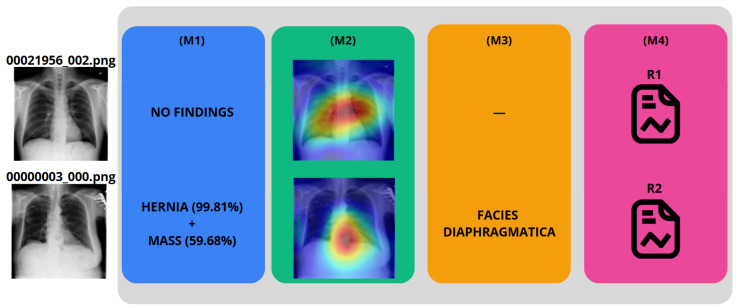
Example of reports generation.

To demonstrate the IHRAS workflow, Figure 6 depicts two example CXR images from the NIH dataset inputted into the system, with the generated reports R1 and R2 presented in Table 7.

The CXR 00021956_002.png is correctly classified as a healthy image, whereas it wrongly attributes a mass diagnosis for 00000003_000.png, although the hernia is correctly identified. Such misdiagnosis is taken into account in Section 4.1.

Additionally, in medical imaging, timely diagnosis is fundamental for effective patient care, particularly in urgent scenarios. Rapid report generation ensures that clinicians receive relevant information without delay. In that regard, IHRAS completes the CXR initial classification in 570 ± 85 ms, generates the GradCAM heatmap generation in 1878 ± 221 ms, performs segmentation in 12,746 ± 713 ms, and generates the medical report generation in 4216 ± 402 ms. These processing times suggest the system’s ability to deliver rapid results.

Since Grad-CAM (M2) visualizations and LLM-generated reports (M4) are conditioned on the model’s predictions (M1), their utility is linked to the quality of those outputs. However, the system’s interpretability components offer importante information regarding model behavior, indicating regions of interest and generating plausible hypotheses that can assist clinicians in decision-making. Rather than presenting these outputs as definitive diagnoses, IHRAS may be used as an assistive tool that complements clinical judgment.

## 5. Conclusions

This work introduced IHRAS, a modular and integrated system designed to enhance the automation and interpretability of chest X-ray analysis by combining disease classification, visual explainability, anatomical segmentation, and structured medical report generation via Large Language Models. IHRAS identifies thoracic pathologies with associated anatomical relevance and produces clinically coherent reports adhering to SNOMED CT standards.

The system demonstrated consistent performance across demographic and clinical subgroups in the NIH ChestX-ray dataset, indicating its potential for equitable clinical deployment. The integration of Grad-CAM for visual explanations and SAR-Net for anatomical localization contributes significantly to transparency and trust in AI-driven radiology.

However, the system’s performance is currently constrained by limitations, including the modest F1-score in the multi-label classification task, which likely arises from the imbalanced data problem, and the lack of evaluation of the Grad-CAM. Hence, as future work, these modules of the IHRAS architecture should be further studied, with the aim of improving its performance, including clinician-based evaluations of the complete system. Future works should also investigate the use of the proposed architecture in other medical imaging applications, such as Computed Tomography scans, Magnetic Resonance Imaging, and ultrasound, or to other anatomical regions beyond the thorax.

## Figures and Tables

**Table 1:** Comparison with related works.

Reference	Year	Classification	Explainability	Segmentation	LLM
[23]	2021	✓	✓	✗	✗
[24]	2022	✗	✗	✓	✗
[25]	2023	✓	✓	✗	✗
[26]	2023	✓	✗	✗	✓
[27]	2024	✓	✗	✓	✗
[28]	2024	✓	✗	✗	✗
Our work	2025	✓	✓	✓	✓

**Table 2:** Radiology Report Instruction Components.

Component	Description
Capacity	You have deep knowledge of clinical report writing using SNOMED CT, based exclusively on the informed findings
Role	Act as a board-certified clinical radiologist preparing an official diagnostic report, with accurate and clear communication of findings
Insight	The model receives the information about the patient age and gender, view position, findings and probabilities, most affected region
Statement	Write a radiological report using SNOMED CT with: Patient Metadata Section, Findings Interpretation, Anatomical Localization
Personality	Professional, concise clinical tone using complete sentences with standard medical report structure
Experiment	Provide a single report

**Table 3:** Comparison of F1-scores across studies.

Reference	Year	Dataset	F1-Score	Precision	Recall
[45]	2021	NIH CXR	0.34	0.28	0.47
[46]	2023	NIH CXR	0.36	0.32	0.43
[47]	2023	MIMIC-CXR	0.27	0.35	-
[48]	2024	Indonesian hospitals	0.30	0.18	1.00
IHRAS’ DenseNet	2025	NIH CXR	0.34	0.25	0.53

**Table 4:** Performance metrics by demographic and clinical characteristics.

Category	Value	Precision	Recall	F1-Score	Specificity
Age	0–24	0.23	0.52	0.32	0.84
	25–49	0.23	0.52	0.32	0.85
	50–74	0.24	0.53	0.33	0.85
	75+	0.24	0.51	0.33	0.86
Gender	M	0.24	0.53	0.33	0.85
	F	0.23	0.52	0.32	0.85
View Position	PA	0.24	0.52	0.33	0.85
	AP	0.23	0.53	0.32	0.85
Findings	No findings	0.24	0.51	0.32	0.85
	Effusion	0.24	0.53	0.33	0.85
	Atelectasis	0.24	0.53	0.33	0.85
	Pneumothorax	0.23	0.52	0.32	0.85
	Edema	0.23	0.54	0.32	0.85
	Infiltration	0.24	0.53	0.33	0.85
	Fibrosis	0.23	0.54	0.32	0.84
	Consolidation	0.23	0.52	0.32	0.85
	Emphysema	0.24	0.51	0.33	0.85
	Mass	0.23	0.53	0.33	0.85
	Pneumonia	0.24	0.55	0.33	0.84
	Hernia	0.27	0.56	0.36	0.85
	Cardiomegaly	0.23	0.54	0.33	0.85
	Pleural Thickening	0.24	0.51	0.32	0.85
	Nodule	0.24	0.51	0.32	0.85

**Table 5:** Maximum Cohen’s h effect sizes across all pairs in categories.

Category	Precision	Recall	F1-Score	Specificity
Age	0.0236	0.0400	0.0214	0.0560
Gender	0.0236	0.0200	0.0214	0.0000
View Position	0.0236	0.0200	0.0214	0.0000
Findings	0.0924	0.1003	0.0845	0.0276

**Table 6:** Medical report evaluation metrics.

Metric	Mean Score	Min Score	Max Score
Faithfulness	0.99	0.89	1.00
Answer Relevancy	0.91	0.75	1.00
Hallucination	0.00	0.00	0.00
Toxicity	0.00	0.00	0.00
Bias	0.00	0.00	0.00
Prompt Alignment	0.86	0.82	0.90

**Table 7:** Medical reports generated from the examples in Figure 6.

Report Section	R1	R2
Metadata	Patient is a 57-year-old male, X-ray obtained in PA projection.	Patient is an 81-year-old female, X-ray obtained in PA projection.
Findings	No acute cardiopulmonary abnormalities are identified. The lungs are clear without evidence of consolidation, pneumothorax, or pleural effusion. The cardiomediastinal silhouette is within normal limits. No osseous lesions or soft tissue abnormalities are detected.	Hernia—Present (high confidence). Mass—Possibly present (moderate confidence). Anatomical Localization: The most affected region is the Facies Diaphragmatica.
Impression	Normal PA chest radiograph without acute abnormalities.	Strong evidence of a hernia involving the abdominal cavity. Suspicion for a mass in the diaphragmatic region, though further evaluation (e.g., cross-sectional imaging or follow-up) is recommended for confirmation. Correlation with clinical history and additional imaging (CT or MRI) may be warranted to further characterize the suspected mass.

## Data Availability

This work uses a publicly available dataset.

## Abbreviations

The following abbreviations are used in this manuscript:

AI	Artificial Intelligence
AP	Antero-Posterior
CFDL	Code-Free Deep Learning
CNN	Convolutional Neural Network
CXR	Chest X-Ray
FL	Federated Learning
Grad-CAM	Gradient-weighted Class Activation Mapping
DenseNet	Densely Connected Convolutional Network
IHRAS	Intelligent Humanized Radiology Analysis System
PA	Postero-Anterior
ResNet	Residual Neural Network
SAR-Net	Structure-Aware Relation Network
SNOMED CT	Systematized Nomenclature of Medicine - Clinical Terms
SVM	Support Vector Machine
XAI	Explainable Artificial Intelligence
